# Loss of Sirt1 Function Improves Intestinal Anti-Bacterial Defense and Protects from Colitis-Induced Colorectal Cancer

**DOI:** 10.1371/journal.pone.0102495

**Published:** 2014-07-11

**Authors:** Giuseppe Lo Sasso, Dongryeol Ryu, Laurent Mouchiroud, Samodha C. Fernando, Christopher L. Anderson, Elena Katsyuba, Alessandra Piersigilli, Michael O. Hottiger, Kristina Schoonjans, Johan Auwerx

**Affiliations:** 1 Laboratory of Integrative and Systems Physiology, Ecole Polytechnique Fédérale de Lausanne, Lausanne, Switzerland; 2 Department of Animal Science, University of Nebraska, Lincoln, Nebraska, United States of America; 3 School of Biological Sciences, University of Nebraska, Lincoln, Nebraska, United States of America; 4 Institute of Animal Pathology, University of Bern, Bern, Switzerland; 5 Institute of Veterinary Biochemistry and Molecular Biology, University of Zurich, Zurich, Switzerland; 6 Institute of Bioengineering, School of Life Science, Ecole Polytechnique Fédérale de Lausanne, Lausanne, Switzerland; Institute of Hepatology - Birkbeck, University of London, United Kingdom

## Abstract

Dysfunction of Paneth and goblet cells in the intestine contributes to inflammatory bowel disease (IBD) and colitis-associated colorectal cancer (CAC). Here, we report a role for the NAD^+^-dependent histone deacetylase SIRT1 in the control of anti-bacterial defense. Mice with an intestinal specific *Sirt1* deficiency (*Sirt1^int−/−^*) have more Paneth and goblet cells with a consequent rearrangement of the gut microbiota. From a mechanistic point of view, the effects on mouse intestinal cell maturation are mediated by SIRT1-dependent changes in the acetylation status of SPDEF, a master regulator of Paneth and goblet cells. Our results suggest that targeting SIRT1 may be of interest in the management of IBD and CAC.

## Introduction

Paneth and goblet cells are highly specialized small intestinal epithelial cells that synthesize and secrete anti-microbial peptides and mucus. These factors represent the first line of defense against pathogens and are essential to maintain the subtle balance among different bacterial species that colonize the mammalian gut [1]. Dysbiotic microbiota impact on the health of the host and contribute to the pathogenesis of several intestinal diseases, such as inflammatory bowel disease (IBD) and colitis-associated colorectal cancer (CAC) [2].

The differentiation and maturation of Paneth and goblet cells is controlled by the Wnt and Notch signaling cascades that cooperate to promote the specification of the different cell lineages [3]. Moreover, the SAM pointed domain containing ETS transcription factor (SPDEF), a downstream effector of both Wnt and Notch pathways, is known to enhance the differentiation of both Paneth and goblet cells from their common progenitor [4]. SPDEF was initially identified as a regulator of the prostate-specific antigen [5], but later also associated to breast, lung, and intestine epithelium, with possible involvement in cancer progression in those tissues [6], [7].

Sirtuin 1 (SIRT1), a NAD^+^-dependent deacetylase [8], is involved in a wide range of cellular processes including metabolism, cell proliferation and apoptosis, and immune response [9]. The role of SIRT1 in the regulation of intestinal homeostasis is only beginning to be elucidated. Recently an involvement of intestinal SIRT1 in systemic bile acid and cholesterol metabolism has been proposed [10]. Furthermore, studies focusing on the role of SIRT1 in colorectal cancer development using Apc^min/+^ mice as a model, showed conflicting results supporting both tumor promoting [11] and tumor suppressing [12], [13] functions.

Using mice with an intestinal-specific *Sirt1* deletion (*Sirt1^int−/−^*), we show here that SIRT1 regulates Paneth and goblet cell maturation and production of anti-bacterial proteins. These effects depend on SIRT1-mediated changes in the acetylation status of SPDEF. Moreover, intestinal *Sirt1* deletion has a major impact on the gut microbiome and protects mice from IBD and CAC. Notably, the effects of *Sirt1* deficiency on the production of anti-bacterial proteins are evolutionary conserved in *C.elegans* highlighting the ancient nature of this function of SIRT1. Taken together our results suggest that targeting SIRT1 may be of interest for the management of IBD and CAC.

## Materials and Methods

### Generation of Sirt1^int−/−^ and Sirt1^int−/−^-LGR5^EGFP-ires-CRE-ert2^ mice

For the generation of *Sirt1* floxed (*Sirt1^L2/L2^*) mice, genomic DNA covering the *Sirt1* locus was amplified from the 129Sv strain by high-fidelity PCR. The resulting DNA fragments were assembled into the targeting vector of the Institut Clinique de la Souris (Strasbourg, France). The construct in which exons 5, 6 and 7 were flanked by LoxP sites was then electroporated into 129Sv embryonic stem (ES) cells (Figure S1B). G418-resistant colonies were selected and analyzed for homologous recombination by PCR and positive clones were verified by Southern blot hybridization. Correctly targeted ES cell clones were injected into blastocysts and transferred into pseudopregnant females, resulting in chimeric offspring that were mated to female C57BL/6J mice that express the Flp recombinase under the control of the ubiquitous cytomegalovirus promoter [14]. Offspring that transmitted the mutated allele and that lost the Flp transgene (*Sirt1^L2/WT^* mice) were then selected, and backcrossed to C57BL/6J mice for ten generations. For the gut microbiota analysis Sirt1^int−/−^ and Sirt1^L2/L2^ were co-housed under specific pathogen free conditions within the same room. Mice treated with AOM/DSS were singly housed after weaning to avoid differences in the DSS intake. However, Sirt1^int−/−^ and Sirt1^L2/L2^ were treated under specific pathogen free conditions within the same room. Notably, Sirt1^int−/−^ and Sirt1^L2/L2^ mice come from the same parents. All animal experiments were done in accordance with institutional and Swiss guidelines and approved by the Cantonal authorities of the Canton of Vaud. Moreover, all animal experiments were conformed to the Swiss Animal Welfare legislation and reviewed by the State Ethical Board of the Canton de Vaud (Animal Welfare Act 2005; Project License N° 2463-2463.1-2605 license to Prof. Johan Auwerx). Finally, all animal experiment were approved by the independent State of Vaud Veterinary office ethical board, which acts in compliance with the Animal Welfare Act, the AAALAC international guidelines, the Swiss and EU legislation. Mice were euthanatized using a brief exposure to CO_2_. This method leads to quick and painless asphyxiation in mice. All the experiments were carried out from October 2011 to April 2014.

### Plasmids

Mammalian expression vector pUSE-SIRT1 was purchased from Upstate. The SPDEF coding sequence belongs to the Mouse Transcription Factor Resource [15]. It was amplified and ligated to the pCDNA3-FLAG or pGEX-GST. pCruzHA SIRT1G261A (plasmid 10963) [16] and pGL2Basic-EcadK1 (plasmid 19290) [17] were purchased by Addgene. pGEX-GST- SPDEFK294Q was generated using site-directed mutagenesis.

### *C.elegans* assays

*C.elegans* strains were grown at 20°C on nematode growth media agar plates (NGM) seeded with *E. coli* strain OP50 unless stated otherwise. Strains used were wild-type Bristol N2, VC199 *sir-2.1(ok434)* IV, SAL129 [*pha-1*(e2123)III;*lys-1*::GFP+*pha-1*(+)], and SAL105 [*pha-1*(e2123)III;*lys-7*::GFP+*pha-1*(+)]. Strains were provided by the *Caenorhabditis* Genetics Center (University of Minnesota). Bacterial feeding RNAi experiments were carried out as described [18]. The *sir-2.1* (R11A8.4) clone was purchased from GeneService and sequenced. *Quantification of GFP* expression was carried out according to described protocols [19]. For picture acquisition of *lys-7*::GFP expression, animals were mounted on 2% agarose pads in 10 mM tetramisole (Sigma) and examined using a Zeiss Axioplan-2 microscope (Carl Zeiss).

### mRNA extraction and RT-qPCR analysis

RNA was isolated from tissues using the TriPure reagent (Roche) according with manufacturer's instructions. cDNA was generated from 1 µg of total RNA using QuantiTect Reverse Transcription Kit (Qiagen). qRT-PCR was carried out using LightCycler 480 SYBR Green I Master Mix (Roche) and analyzed through ΔΔCT calculation. Values were normalized to Cyclophilin expression. Primers are listed in Table S1 in File S1.

### Luciferase assay

PC3 cells (ATCC) in 96/wells plates were co-transfected with a reporter containing the SPDEF response element of the human E-cadherin promoter, pGL2Basic-EcadK1 and pcDNA3, pCDA3-FLAG-SPDEF or pCDA3-FLAG-SPDEFK294Q with or without pUSE-SIRT1 expression vectors (jetPEI transfection; Polyplus). Media was removed after 24 hr, cells washed with cold PBS, and Luciferase Substrate (Bright-Glo Luciferase Assay System, Promega) was added before Luciferase measurement by the Victor ×3. β-galactosidase was used for normalization.

### In vitro acetylation and deacetylation assays

In vitro acetylation and deacetylation assays were performed as originally described [20]. Briefly, 1 µg of recombinant SPDEF protein, obtained from the BL21 strain, were incubated with 500 ng of recombinant p300 in acetylation buffer (50 mM Tris-HCl, pH 8, 100 mM NaCl, 10% glycerol, 1 mM PMSF, 1 mM DTT, 1 µg/ml bepstatin, 1 µg/ml leupeptin, 1 µg/ml pepstatin, 1 mM sodium butyrate and 150 µM acetyl-CoA) for 1 hour at 30°C. After incubation, samples were resolved on SDS-PAGE and analyzed by western blot or used for in vitro deacetylation assays. For deacetylation assays, 1 µg of acetylated SPDEF was incubated with 500 ng recombinant SIRT1 protein in deacetylation buffer (50 mM Tris-HCl, pH 9, 4 mM MgCl2, 0.2 mM DTT, 1 µg/ml bepstatin, 1 µg/ml leupeptin, 1 µg/ml pepstatin, and 1 mM NAD^+^) for 30 minutes with constant agitation. The incubated samples were resolved on SDS-PAGE and analyzed by western blot or used to map an acetylated residue with nano-LC-MS/MS. Recombinant p300, SIRT1, SIRT6, and SIRT7 proteins were made by Dr. Michael O. Hottiger (University of Zurich). Cell based deacetylation was assayed in Hek293T cells transfected by pCDA3-FLAG-SPDEF or pCDA3-FLAG-SPDEFK294Q with or without pUSE-SIRT1 or pCruzHA-SIRT1G261A expression vectors by jetPEI Transfection kit (Polyplus). 22 hours later, media was replaced with fresh media (Dulbecco's modified Eagle's medium with 4.5 g/l glucose, 10% fetal calf serum, 0.1 mM NEAA, 50 µg/ml gentamicin and 1 mM sodium butyrate). After 2 hours, whole cell-lysates were prepared using IP lysis buffer (50 mM Tris HCl, pH 7.4, 150 mM NaCl, 1 mM EDTA, 1% TRITON X-100, 10 mM sodium butyrate and 10 mM nicotinamide) supplemented with Complete Protease Inhibitor Cocktail Tablets (Roche). 0.5 mg to 1 mg of whole cell-lysates was used for immunoprecipitation (IP). IP was performed with FLAG-Agarose Affinity Gel as described by the supplier (A2220, Sigma-Aldrich). After IP, samples were applied to SDS-PAGE and analyzed by western blotting.

### Cell culture, transfection, and antibodies

HEK293 and PC3 cells were cultured in Dulbecco's modified Eagle's medium including 4.5 g/l glucose, 10% fetal calf serum, 0.1 mM NEAA and 50 µg/ml gentamicin at 37°C under a 5% CO_2_ atmosphere. HEK293T and PC3 cells were transfected using JetPei reagent (*Polyplus Transfections*, Illkirch, France) according to the manufacturer's instructions. Antibodies: Lysozyme (Abcam, Ab36362), ChRA (Santa Cruz, sc-13090), Acetylated lysine (Cell signaling, 9441L), Sirt1 (Abcam, ab12193), β-catenin (Sigma, A5441), L-FABP (Santa Cruz, sc-50380), PCNA (Santa Cruz, sc-56), HSP90 (BD Transduction Laboratories, 610418), anti-FLAG (Sigma, F1804), Tubulin (Santa Cruz, sc-5286), SPDEF for western blot (Sigma, AV32533), SPDEF for IHC was kindly provided by Prof. J. Whitsett.

### Subcellular fractionation

Cytoplasm and nucleoplasm fractions were obtained from *Sirt1^L2/L2^* and *Sirt1^int−/−^* isolated crypt. Crypts were isolated following a published protocol [21]. Single crypt-derived cells were finally washed with ice-cold PBS, and incubated in buffer A (10 mM KCl, 1.5 mM MgCl2, 0.5 mM DTT10 mM, 10 HEPES-KOH, pH 7.4) including a proteinase inhibitor cocktail (Roche) for 5 min. After 50 strokes in a dounce homogenizer, cytoplasm fraction was collected by centrifugation (1.4 k× g for 5 min, 4°C). Pellets were washed two times with buffer A. Nuclei were incubated in buffer B (150 mM NaCl, 0.1% NP-40, 50 mM Tris-HCl, pH 7.4) containing a proteinase inhibitor cocktail for 30 min on ice. Nucleoplasm was collected by centrifugation (2 k× g for 5 min, 4°C). Proteins were quantified using the Lowry method and processed for western blot assay.

### GFP^+^ cells analysis

Crypts from *Sirt1^L2/L2^-* and *Sirt1^int−/−^-LGR5^EGFP-ires-CRE-ert2^* intestines were isolated as previously described (see *Subcellular fractionation* paragraph) [22]. Single cells were analyzed by FACS to detect the GFP^+^ cells.

### Fractionation of cells along the crypt–villus axis

The sequential isolation of mouse small intestinal epithelial cells along the crypt–villus axis was performed as described previously [23] with few modifications. Briefly, the entire small intestine (duodenum to terminal ileum) was removed, flushed, and cut in small pieces (2–5 mm) and incubated at 37°C for 15 min in 15 ml of buffer A (96 mM NaCl, 1.5 mM KCl, 27 mM Na-citrate, 8 mM KH_2_PO_4_, and 5.6 mM Na_2_HPO_4_, pH 7.3). Then it was incubated for 10 min in 15 ml of buffer B (PBS plus 1.5 mM EDTA, 0.5 mM dithiothreitol, and 1 mg/ml bovine serum albumin) in a shaking 37°C incubator. At the completion of the 15-minute incubation, detached enterocytes were collected (Fraction 1) and 15 ml of fresh buffer B added to the tissue. This procedure was repeated four more times, the steps lasting 25, 25, 25 and 30 min, respectively (Fractions 2, 3, 4 and 5), for a total of 120 min of incubation time. At the completion of the final incubation period, cells collected from each of the 5 fractions were harvested by centrifugation at 1500 rpm at 4°C for 5 min. Cell pellets were washed twice and lysed in RIPA buffer. Alkaline phosphatase activity was assayed by Alkaline Phosphatase Assay kit (BioVision).

### Mass spectrometry analysis

Gel lanes were cut into pieces and subjected to in-gel digestion with endoproteinase Glu-C or trypsin. Peptide digests were resuspended and analyzed by nano-LC-MS/MS using an Orbitrap Elite mass Spectrometer (Thermo Fischer Scientific) coupled to an ultraperformance LC (UPLC) system (Thermo Fischer Scientific Ultimate 3000 RSLC). Data analysis was performed with Proteome Discoverer (v. 1.3) and searches were performed with Mascot and Sequest against a mouse database (UniProt release 2013_01). Data were further processed, inspected and visualized using Scaffold 3 software.

### *In situ* hybridization

The *in situ* probes used in this study correspond to expressed sequence tags or fully sequenced cDNAs obtained from Open Biosystems. The accession numbers for these probes are as follows: mouse *Olfm4* BC141127 (9055739), mouse *Defa4* BC134360 (40134597). To ensure the specificity of the probes, we generated both sense and antisense probes by *in vitro* transcription using DIG RNA labeling mix (Roche) according to the manufacturer's instructions and to published methods [24]. ISH was done using the fully automated instrument Ventana XT (Roche). Chemicals were from Roche Diagnostics. Briefly, formalin fixed paraffin-embedded sections were de-paraffinized and rehydrated and pretreated by enzymatic digestion (protease1, 4 min at 37°C). Hybridization was performed adding to each slide 50 or 100 ng of the probe diluted in Ribohybe at 65°C for 6 hours.

### Bactericidal assay

Bactericidal assays were carried out as described [25] with few modifications. Briefly, the small intestine of adult mice was rinsed with ice-cold PBS and segments incubated in 30 mM EDTA to elute epithelial cells. Total proteins from the epithelial cells were extracted using NP40 lysis buffer (20 mM Tris pH 7.4, 150 mM NaCl, 2.5 mM KCl, 5 mM EDTA, 5% [vol/vol] glycerol, 1% [vol/vol] NP40) supplemented with complete protease inhibitor. 100 µg of proteins were incubated for 60 minutes at 37°C in 10 mM iPIPES buffer containing exponentially growing *E.coli* K12 (ATCC, 10^6^ CFU/mL). 20 µL of sample was diluted and plated in LB-agar solid medium. Surviving bacteria were quantified as CFU on plates after overnight incubation at 37°C. Bacteria incubated in iPIPES without added proteins were considered as controls.

### Colitis and Colitis-associated colorectal cancer models

DSS-induced colitis was induced as previously described [26]. Daily changes in body weight were assessed. Rectal bleeding was scored on a scale from 0 to 5, indicating no (0) or highly severe (5) rectal bleeding. Ilea and colons were snap-frozen or fixed with 4% Forma-Fixx (Thermo scientific) and embedded in paraffin. *In vivo* intestinal permeability was examined in mice as described [27]. Colitis-induced colorectal cancer (AOM/DSS model) was induced as previously described [28]. After sacrifice, mouse colons were opened longitudinally and after 2 hours fixation in 4% Formal-Fixx, briefly stained with Coomassie Blue to visualize tumors. Tumors were counted in a blinded fashion by a pathologist. Tumors size was assayed by gauge. Small pieces of terminal Ilea and proximal colons were snap-frozen and the rest fixed with 4% Forma-Fixx (Thermo scientific) and embedded in paraffin.

### Statistical analysis

Data were checked with the Shapiro–Wilk test for normality in R prior to performing significance tests. Variables with a W value ≥0.80 were considered as approximately normally distributed. Two variable comparisons were calculated using Welch's two-tailed *t*-test. For multiple comparisons, Bartlett's test was performed to check for equal variance (p>0.10) and then one-way ANOVA was performed with the Bonferroni post-hoc test. Variables with a Shapiro-Wilk W value <0.80 were considered as non-normal and comparisons were calculated using the Wilcoxon signed-rank test. The Kaplan-Meier method was used for the survival analysis in worms. Data are expressed as mean ± SEM, and for all significance comparisons, *p* values smaller than 0.05 were considered as statistically significant. ^*^p<0.05; ^**^p<0.01; ^***^p<0.001. This study had an exploratory nature and there was no pre-specified effect size. The sample size was chosen based on the study feasibility and potential statistical power. Sample sizes are consistent with those reported in similar studies. Statistical analysis for the gut microbiota analysis is reported in the Supplemental Information.

## Results

### *Sirt1* deletion increases anti-microbial response in mammals and nematodes

**Figure 1:**
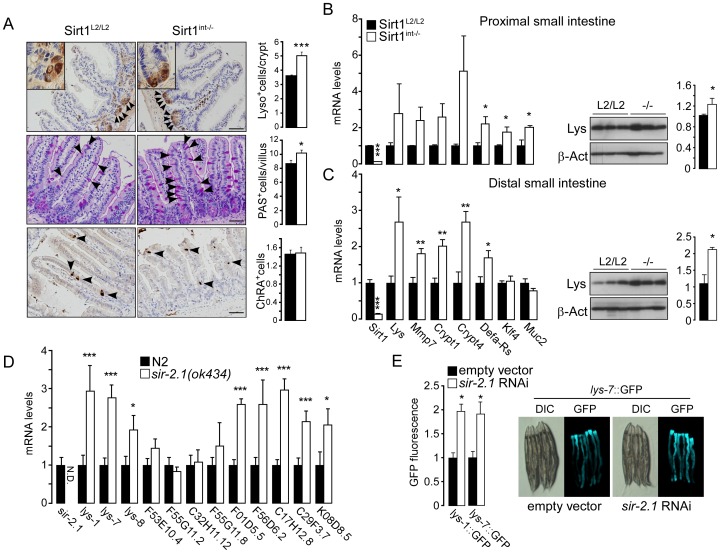
SIRT1 deletion increases Paneth and goblet cell number and up-regulates anti-bacterial gene expression in mice and worms. **A**, Representative images of Paneth (Lysozyme^+^ cells), goblet (Periodic acid-Shiff^+^ cells), and enteroendocrine (Chromogranin A^+^ cells) cell staining. (N = 3 mice; 5–10 field per mouse, 50–100 crypt/villi per field). Bar = 50 µm. **B–C**, mRNA and protein levels of Paneth (*Lys, Mmp7, Crypt1, Crypt4, Defa-Rs*) and goblet (*Klf4, Muc2*) markers are increased in the proximal and distal small intestine of *Sirt1^int−/−^* mice (N = 6–8). For RTqPCR analysis, *rps12* was used as reference gene. β-Actin was used as loading control in the western blot. **D**, Increased expression of genes involved in pathogen defense in the *sir-2.1* (*ok434*) *C. elegans* mutant (N = 6; each N means a single population of ∼500 worms). *Act1* and *Y45* were used as reference. **E**, *sir-2.1* siRNA induces *lys-1* and *lys-7* driven GFP expression in *lys-1::GFP* and *lys-7::GFP* reporter worms. In the same figure a representative image of *lys-7::GFP* before and after *sir-2.1* siRNA is shown (DIC, differential interference contrast). Results are expressed as mean±SEM. *P<0.05; **P<0.01; ***P<0.001.

We first determined the localization of SIRT1 protein along the villus/crypt axis. Since SIRT1 appears highly enriched in the small intestine crypts (Figure S1A), we bred Villin-Cre transgenic mice with mice in which exons 5–7 of the *Sirt1* gene were flanked by LoxP sites (*Sirt1^L2/L2^*) to generate intestinal-specific *Sirt1* knockout mice (*Sirt1^int−/−^*) (Figure S1B–C). Unlike a previously reported deletion of *Sirt1* exon 4 [29], no truncated and potentially active SIRT1 fragments were detected in *Sirt1^int−/−^* mice (Figure S1C). *Sirt1^int−/−^* intestines showed a significant increase in the number of Paneth (lysozyme^+^ and Defa4^+^) and goblet (PAS^+^), but not of enteroendocrine (ChRA^+^) cells (Figure 1A, Figure S1D). Accordingly, mRNA levels of Paneth (*Lys, Crypt1, Crypt4, Defa-Rs*) and goblet (*Klf4, Muc2*) cell markers, as well as lysozyme protein abundance and *Defa4* mRNA detected through *in situ* hybridization, were induced in the proximal and distal intestine of *Sirt1^int−/−^* mice (Figures 1B–C, Figure S1D–E).

We next evaluated whether *Sirt1* is correlated to the genes involved in anti-bacterial defense using the BXD mouse genetic reference population (www.genenetwork.org) [30]. As no intestinal gene expression data are available, we analyzed hematopoietic cells, which are part of the arsenal of cells that protect against pathogens. In line with our observations, *Sirt1* expression correlates negatively with the expression of several Defensin-related genes (Figure S1F).

To test whether the link between *Sirt1* and the anti-bacterial defense was evolutionary conserved, we used *C. elegans*. Remarkably, the expression of a wide range of genes involved in anti-microbial defense (*lyz-1, lyz-7, lyz-8*) [31], as well as that of genes induced by specific pathogens (F01D5.5, F56D6.2, C17H12.8, C29F3.7, K08D8.5) [32] was enhanced in *sir-2.1* [33] mutant worms (Figure 1D). Furthermore, in *lys-1::GFP* and *lys-7::GFP* reporter strains [31], s*ir-2.1* siRNA significantly induced both *lys-1*- and *lys-7*-dependent GFP expression (Figure 1E). These data hence indicate that the effect of *Sirt1* in the anti-microbial response is evolutionary conserved from mammals to worms.

### Intestinal *Sirt1* deletion impacts on gut microbiota

**Figure 2:**
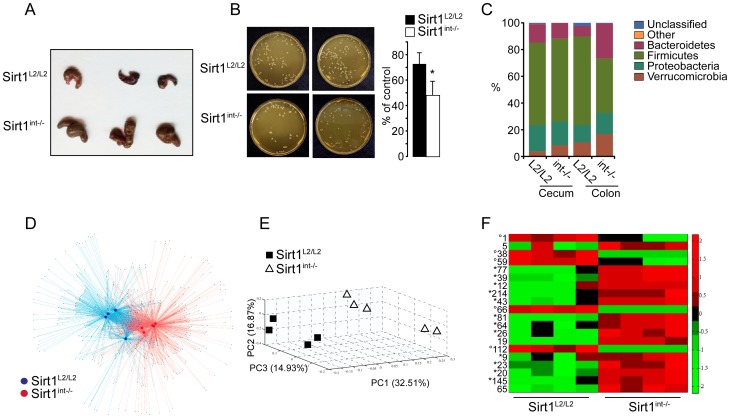
Intestinal deletion of *Sirt1* rearranges the gut microbiome. **A,** Representative image showing a significant increase in cecum size in *Sirt1^int−/−^* mice. **B,** Bactericidal capacity of small intestine (duodenum) in *Sirt1^int−/−^* and *Sirt1^L2/L2^* mice was assayed by colony forming unit assay. Results are expressed as % of the control (no proteins) (N = 7). **C,** Distribution of microbial Phyla in colon tissue and cecum contents *of Sirt1^L2/L2^* and *Sirt1^int−/−^* mice. **D,** Network-based analyses of cecal bacterial communities in *Sirt1^L2/L2^* and *Sirt1^int−/−^* mice. Each large circle represents an animal and each smaller dot represents an OTU; *Sirt1^L2/L2^* (Blue) and *Sirt1^int−/−^* (Red). **E,** Principal Coordinate Analysis (PCA) (PERMANOVA p = 0.012, and ANOSIM p = 0.007) showing significant separation of the microbial communities between *Sirt1^L2/L2^* and *Sirt1^int−/−^* mice. **F,** Heatmap showing the top 20 different OTUs between *Sirt1^L2/L2^* and *Sirt1^int−/−^* mice. The abundance values were log transformed and standardized and plotted within matlab. *indicates *Barnesiella*, *Bacteroides*, and *Prevotella*; **°**indicates *Clostridum* genus (see also Table S3 in File S1). Results are expressed as mean±SEM. *P<0.05; **P<0.01; ***P<0.001.

Considering the fundamental role that Paneth and goblet cells play in protecting the mammalian gut from aberrant bacterial colonization, we next analyzed the impact of a *Sirt1* deletion on gut microbiota. *Sirt1^int−/−^* intestines did not only have a larger cecum (Figure 2A), indicative of a modified microbiome [34], they also had a higher bactericidal capacity (Figure 2B). Using DNA extracted from either the intraluminal cecum content or colon tissue, we amplified the V3 region of the bacterial 16S rRNA gene. After removal of low quality reads and all singleton Operational Taxonomic Units (OTUs) (Table S2 in File S1), 98% of the reads fell into the Core Measurable Microbiome (CMM–see methods). The microbial community was dominated by 4 phyla, *Firmicutes, Bacteroidetes, Proteobacteria, and Verrucomicrobia* (Figure 2C) [35]. To map the microbial community composition and structure across the *Sirt1* mutation we used an OTU-based method. In network-based analyses, the cecum microbial communities of *Sirt1^L2/L2^* and *Sirt1^int−/−^* mice highlighted community differences between genotypes (Figure 2D), an observation further supported by Principal Coordinates Analysis (PCA; Figure 2E). Indicator microbial species that contribute towards community differences between *Sirt1^L2/L2^* and *Sirt1^int−/−^* mice were identified and OTUs displayed in Figure 2F and Table S3 in File S1. Interestingly, a majority of enriched OTUs in *Sirt1^int−/−^* mice were of the genus *Barnesiella*, *Bacteroides*, and *Prevotella* (phylum *Bacteroidetes*), found in the normal human and mouse gut, which are notably decreased in IBD [36], [37]. In contrast, the genus *Clostridium* (phylum *Firmicutes*) was expanded in *Sirt1^L2/L2^* mice (Figure 2F and Table S3 in File S1). These results show that intestinal *Sirt1* deletion and the consequent changes in Paneth and goblet cells modify the gut microbiome.

### Intestinal *Sirt1* deletion protects from colitis and colitis-induced colorectal cancer

**Figure 3:**
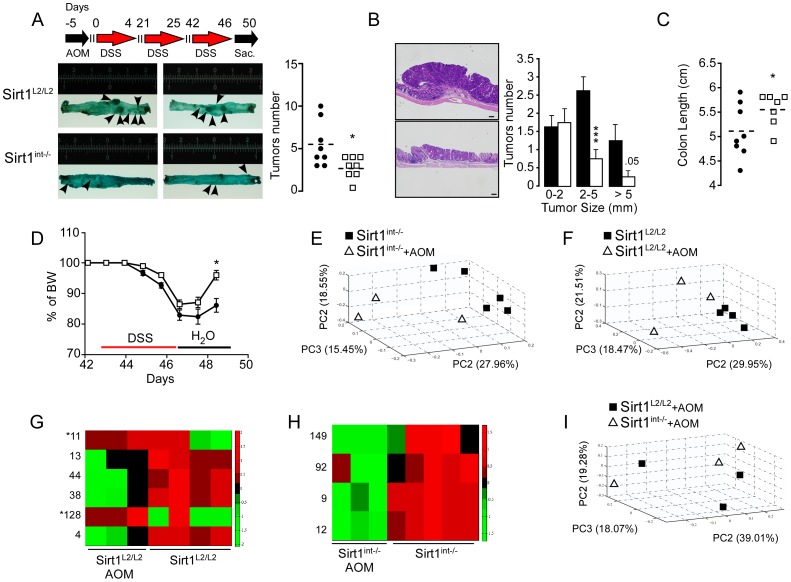
Intestinal *Sirt1* deletion impacts on the development of colorectal cancer. **A–B,** Schematic representation of the AOM/DSS protocol (top left panel) and representative image of colons from *Sirt1^int−/−^* and control mice after CAC induction (Bar = 200 µm) (bottom left panel). *Sirt1^int−/−^* mice show significantly less tumors (right panel). **B**, Representative picture of a colon section from a *Sirt1^int−/−^* and control mouse after CAC induction (left panels). Tumor size (right panel). **C,** Colon length at the time of sacrifice (AOM/DSS). **D,** Percentage of body weight change. For the AOM/DSS experiment 8 mice for each genotype were used. *P<0.05; **P<0.01; ***P<0.001. **E–F,** Principal Coordinate Analysis (PCA) of bacterial sequences from colon tissue performed using unweighted UniFrac distance matrix. **E**, *Sirt1^int−/−^* colon tissue before and after AOM treatment (PERMANOVA p = 0.003, ANOSIM p = 0.016). **F**, *Sirt1^L2/L2^* colon tissue with and without AOM treatment (PERMANOVA p = 0.067, ANOSIM p = 0.038). **G–H,** Most statistically significant OTUs before and after AOM in both *Sirt1^L2/L2^* (**G**), and *Sirt1^int−/−^* (**H**), colon tissues; *Indicates *Helicobacter* and *Desulfovibrio* (see also Table S4 in File S1). **I,** PCA of bacterial sequences from colon tissue after AOM (PERMANOVA p = 0.767, ANOSIM p = 0.167).

Given these last observations, we studied the impact of *Sirt1* on the pathogenesis of both colitis and CAC. *Sirt1^int−/−^* mice, exposed to dextran sulfate sodium (DSS), showed a milder colitis characterized by lower body weight loss, bleeding score, a trend towards reduced intestinal permeability, and a decreased expression of inflammatory genes (Figure S2A–D). These results incited us to study how *Sirt1^int−/−^* mice react to CAC. Mice were hence injected with the AOM (azoxymethane) carcinogen before a subsequent exposure to three cycles of DSS (Figure 3A). Remarkably, the number and size of tumors in *Sirt1^int−/−^* intestines were smaller (Figures 3A–B). The better health status of *Sirt1^int−/−^* mice was further underlined by the longer colon length and faster body weight recovery after the last DSS cycle (Figures 3C–D).

Analysis of the microbial community after DSS/AOM exposure revealed significant changes in *Sirt1^int−/−^* mice (Figure 3E), while the change was smaller in *Sirt1^L2/L2^* mice (Figure 3F). Indicator microbial species, before and after DSS/AOM, were identified (Figures 3G–H and Table S4 in File S1). Of note, OTUs belonging to genus *Helicobacter* and *Desulfovibrio* were only increased after DSS/AOM in *Sirt1^L2/L2^* mice (Figure 3G and Table S4 in File S1). Interestingly, although the role of *Helicobacter* in colorectal cancer remains controversial [38], *Desulfovibrio* is considered to take part in the pathogenesis of ulcerative colitis and Crohn's disease [39]. Microbial communities in *Sirt1^L2/L2^* and *Sirt1^int−/−^* mice after DSS/AOM treatment were not significantly different (Figure 3I). Altogether, we hypothesize that changes in the general microbial community under normal conditions (Figure 2), as well as in specific genera after DSS/AOM, could determine the different susceptibility of the two genotypes to colitis and CAC.

### Hyper-acetylated SPDEF drives Paneth and goblet cell maturation upon intestinal-specific *Sirt1* deletion

To understand the molecular changes underlying these prominent phenotypes, we explored different possible pathways that contribute to Paneth and goblet cell development and maturation. Since the intestinal cell types differentiate from intestinal stem cells (ISC) [3], we first evaluated whether *Sirt1* deletion might impact the ISC population. *In situ* hybridization using RNA probes for *Olfm4*, an ISC marker, failed to show differences in number and localization of ISC between *Sirt1^int−/−^* and *Sirt1^L2/L2^* mice (Figure S3A). By crossing *Lgr5^-EGFP-IRES-CreERT2^* [40] with *Sirt1^int−/−^* or *Sirt1^L2/L2^* mice, we generated a mouse line with an intestinal-specific *Sirt1* deletion, which concomitantly expresses GFP in ISCs. FACS analysis of GFP-positive cells (Figure S3B), as well as confocal imaging of crypts (Figure S3C), and gene expression profiling (Figure S3D–E), revealed no differences in ISC number.

We then analyzed the molecular pathways involved in intestinal cell commitment. The Wnt/β-catenin signaling pathway is one of the main driving forces of intestinal cell differentiation. Of note, the relationship between β-catenin and SIRT1 has been extensively studied in both mouse and cell lines, producing conflicting results [11], [12]. Neither the number nor the localization of proliferative cells upon BrdU incorporation was different between *Sirt1^int−/−^* and *Sirt1^L2/L2^* mice (Figure S4A). Accordingly, villi and crypt lengths were indistinguishable (Figure S4B). Furthermore, no SIRT1-dependent modulation of β-catenin levels or localization could be observed by β-catenin immunostaining and protein fractionation from crypts (Figure S4C–D). Thus, in our model nothing heralds a possible involvement of β-catenin.

**Figure 4:**
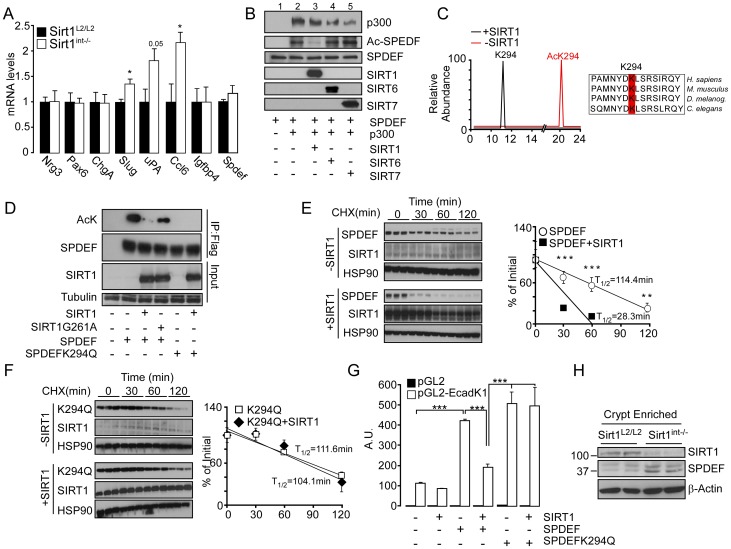
Hyper-acetylation by SIRT1 stabilizes SPDEF and triggers Paneth and goblet cells maturation. **A,** SPDEF target genes (*Slug, uPA, Ccl6*) are induced in *Sirt1^int−/−^* intestines. GIF1 (*Nrg3, Pax6, ChgA*) and SOX9 (*Igfbp4*) target genes, as well as *Spdef* are not changed. **B,** In vitro acetylation/deacetylation assays demonstrates that p300 acetylates SPDEF and SIRT1, but not SIRT6 and SIRT7, deacetylates SPDEF. **C**, Nano-LC-MS/MS shows SIRT1-dependent in vitro deacetylation of AcK294 (left panel). Sequence alignment showing the evolutionary conserved K294 residue (right panel). **D,** SIRT1, but not SIRT1G261A, deacetylates SPDEF in HEK293 immunoprecipitates. The SPDEFK294Q mutant is not acetylated. Tubulin was used as loading control. **E–F** SPDEF, SPDEFK294Q, and SIRT1 were transfected in the HEK293 cell line and visualized by immunoblotting before (0 min) and after cycloheximide (CHX) treatment. HSP90 is used as loading control (left panels in E and F). The relative stability of SPDEF or SPDEFK294Q was calculated by ImageJ (right panels in E and F). **G,** PC3 cells were co-transfected with an E-Cadherin promoter luciferase reporter construct, wild type or SPDEFK294Q in presence or absence of SIRT1. SIRT1 represses SPDEF-dependent reporter activation. The acetylated-mimic SPDEFK294Q mutant is constitutively activate and not affected by SIRT1. **H**, Immunoblotting of crypt enriched fractions from *Sirt1^int−/−^* and *Sirt1^L2/L2^* mice show increased SPDEF protein levels in *Sirt1^int−/−^* intestines. β-Actin was used as loading control. Results are expressed as mean±SEM. *P<0.05; **P<0.01; ***P<0.001.

Paneth and goblet cells share also other transcription factors downstream from the Notch and Wnt signaling cascades, such as GIF1, SPDEF, and SOX9, which are required for their differentiation [4], [41], [42]. Thus, we compared the expression of their target genes in *Sirt1^int−/−^* and control small intestines. Of note, only SPDEF target genes, *Slug*, *uPA*, and *Ccl6* [4], [7], were induced, but GIF1 (*Nrg3, Pax6, ChgA*) and SOX9 (*Igfbp4*) targets were unchanged (Figure 4A), hinting to a possible involvement of SPDEF. Since *Spdef* mRNA levels were not different between *Sirt1^int−/−^* and *Sirt1^L2/L2^* mice (Figure 4A), we tested whether SIRT1 alters SPDEF activity by modifying its acetylation status. Among the three most important acetyltransferases, GCN5, P/CAF, and p300, only the latter robustly acetylated SPDEF *in vitro* (Figure 4B, lane 2). Interestingly, the observed SPDEF acetylation was reversed by SIRT1 but not by the other nuclear sirtuins, such as SIRT6 and SIRT7 (Figures 4B, lanes 3–5). Nano-LC-MS/MS analysis identified the evolutionary conserved lysine residue K294 as the site that was deacetylated by SIRT1 (Figure 4C). We then tested the SIRT1-dependence of SPDEF deacetylation in HEK293T cells co-transfected with SIRT1 or SIRT1G261A [16], a catalytically inactive SIRT1 mutant. We also used a mutant of SPDEF protein, in which the K294 lysine residue was replaced by a glutamine (SPDEFK294Q), mirroring, at least in the charge, the hyperacetylated status of SPDEF (Figure 4D). While SIRT1 was able to fully deacetylate the wild type SPDEF, SIRT1G261A only minimally affected SPDEF acetylation. Furthermore, the SPDEFK294Q mutant was not acetylated, confirming that the K294 is the key acetylation site (Figure 4D).

To understand how the acetylation status could affect SPDEF activity, we assayed both wild type and SPDEFK294Q in protein stability assays. Remarkably, in presence of cycloheximide, wild type SPDEF deacetylation by SIRT1 significantly reduced its stability (Figure 4E), while that of the SPDEFK294Q mutant was not affected (Figure 4F). This change in the SPDEF protein stability also influenced SPDEF transcriptional activity. Hence, the presence of SIRT1 reduces the transcriptional activity of wild type SPDEF on a cognate target reporter [17], but not that of the SPDEFK294Q, which seems constitutively active (Figure 4G). For technical reasons it was impossible to immune-precipitate SPDEF and determine its acetylation status *in vivo* from mouse intestines, but total protein quantification by western blot showed an increase in the amount of total SPDEF in *Sirt1^int−/−^* mice (Figure 4H). SIRT1-dependent deacetylation of SPDEF hence reduces both its stability and transcriptional activity. We can therefore assume that SIRT1 deletion and the consequent SPDEF hyperacetylation, stabilizes the SPDEF protein and increases its transcriptional potential, ultimately favoring Paneth and goblet cells maturation.

## Discussion

To bypass the pleiotropic effects of SIRT1 and focus on its intestine-specific role, we generated a new intestine-specific *Sirt1* mutant mouse model. Intestinal *Sirt1* knockout mice have been previously generated [10], [11] through conditional deletion of *Sirt1* exon 4, which encodes the catalytically active domain of the protein. This strategy generates a truncated SIRT1 protein [29], which has lost its enzymatic activities but still interacts with other proteins, potentially confounding the interpretation of results. Our model, in contrast, results in the full absence of SIRT1 in the intestine, eliminating these possible confounders. The characterization of this new *Sirt1^int−/−^* mouse line reveals an evolutionary conserved process in which *Sirt1* (or *Sir-2.1*) inactivation protects the intestine from pathogens. This effect is, at least partially, mediated by the hyperacetylation of SPDEF, which we identify as a new SIRT1 deacetylation target. Acetylation stabilizes the SPDEF and increases its capacity to enhance Paneth and goblet cells differentiation and maturation, ultimately enhancing the intestinal anti-bacterial potential and remodeling the gut microbiota.

Recently, an SPDEF-dependent increase of intestinal goblet cells together with the inhibition of progenitor cells proliferation, and a reduction in Paneth and enteroendocrine compartment has been reported in a doxycycline-dependent intestinal-specific SPDEF over-expressing mouse model [7], [43]. These data, however, are in apparent contrast to the original characterization of the SPDEF knockout mouse model [4], in which SPDEF deficiency was responsible for both Paneth and goblet cells depletion, without affecting epithelial cell proliferation. Moreover, doxycycline treatment is known to heavily affect gut microbiota [44], hence potentially affecting Paneth and goblet cells function and maturation. Thus, the SPDEF-dependent increases of Paneth and goblet cells observed in our model (*Sirt1^int−/−^* mice) seems to fit better with the original discovered role of SPDEF [4].

Furthermore, together with the Paneth and goblet cell number, key antimicrobial peptides, such as lysozyme and the cryptidines, are increased in the intestine of *Sirt1^int−/−^* mice. The induction of these genes and proteins enhances the bactericidal capacity of *Sirt1^int−/−^* crypt content, and most likely underlies the rearrangement of the gut microbiome that we observed. SIRT1 in the intestine was recently suggested to control ileal bile acid absorption and as such impacts on systemic bile acid homeostasis [10]. In our study, the expression of genes involved in bile acid transport and sensing was, however, not altered, making changes in bile acids an unlikely contributor to the altered gut flora (Figure S1E).

Importantly, our *Sirt1^int−/−^* mice develop fewer tumors and show milder inflammation after AOM/DSS. Previous studies on the role of *Sirt1* in colorectal cancer have used mice, in which the exon 4 of *Sirt1* was deleted [11] or in which *Sirt1* was overexpressed in the intestine [12]. The outcome of these studies showed both tumor promoting and inhibiting effects related to *Sirt1* [11], [12]. Besides the fact that we used a different *Sirt1^int−/−^* mouse model, another notable difference between our work and these studies is that they use Apc^min/+^ mice. Apc^min/+^ mice are representative of hereditary and rare forms of colorectal cancer, based on mutations in the APC gene. The sporadic form of colorectal cancer, i.e. CAC, commonly found in the general population, however, is better represented by the AOM/DSS mouse model. These previous studies using the APC^min/+^ mouse model furthermore showed conflicting results regarding the involvement of the Wnt/β-catenin pathway. The reduction of polyp number in a *Sirt1* over-expressing APC^min/+^ mouse model was in one study attributed, to the inhibition of β-catenin, a downstream target of the Wnt signaling [12], whereas another study suggested that SIRT1 promotes the Wnt signaling both *in vitro* and *in vivo* [11]. Our mouse model did not show differences in intestinal cell proliferation, β-catenin activation/inhibition, and intestinal stem cells involvement (Figure S3 and S4). However, a growing body of evidence indicates that the possible role of SIRT1 in the Wnt signaling is complex and tightly related to possible indirect mechanisms which involve other proteins, as well as differences in cellular context [45], [46], [47], [48].

Our results hence may be relevant for a large number of subjects suffering from various forms of IBD and CAC and may open a potential perspective to treat these conditions in which the gut microbiota is involved by targeting SIRT1.

## Supporting Information

Figure S1 **Generation and characterization of** ***Sirt1^int−/−^*** **mice.** **A,** Villus/crypt fractionation shows a decrease in the alkaline phosphatase (ALP) activity from the top of the villus (F1) to the bottom (crypt, F5). SIRT1 shows a gradient increasing from the top to the bottom of the villus/crypt unit. PCNA and LFABP, respectively a proliferative and a differentiation marker, showed opposite protein distribution, confirming the validity of the fractionation protocol. Tubulin is the loading control. **B,** Schematic graph of the gene targeting strategy of exons 5–7 of the *Sirt1* gene. **C** Western blot analysis of SIRT1 expression in intestine and liver of *Sirt1^int−/−^* and control mice showing the tissue-specific deletion of SIRT1. *Non-specific band. β-Actin is the loading control. **D,** *In situ* hybridization using *Defa4* RNA probe to detect Paneth cells in *Sirt1^int−/−^* and *Sirt1^L2/L2^* mice. **E**, RTqPCR analysis of bile acids transport and sensing mRNAs in proximal and distal small intestine of *Sirt1^L2/L2^* and Sirt1^int−/−^ mice (N = 6–8 mice). For RTqPCR analysis *rps12* is used as reference. **F,** inverse correlation between expression of *Sirt1* (y-axis) and mRNAs of each indicated defensin-related gene (x-axis) in hematopoietic cells of BXD mice strains (N = 22). Results are expressed as mean±SEM.
(TIF)

Figure S2 **Intestinal** ***Sirt1*** **deletion impacts on the development of colitis.** **A,** Percentage of body weight loss observed during 7 days of 2% DSS treatment. **B,** Rectal bleeding score. *Sirt1^int−/−^* mice show significant less weight loss and a reduced bleeding score compared with wild type mice. Scoring details are in Supplemental Materials & Methods. ANOVA statistical analysis with Bonferroni post-hoc test was performed for each time point. **C,** *In vivo* intestinal permeability measurement. *Sirt1^int−/−^* mice show a reduced FITC-derived fluorescence in the blood suggesting less permeability, consequence of less inflammation. **D,** RT-qPCR analysis of *lys*, *Crypt1*, *Tnfα*, and *Mcp1* mRNAs in the distal ileum of *Sirt1^int−/−^* and *Sirt1^L2/L2^* after 2% DSS. *Cyclophilin* is used as reference. For the colitis experiment, 8 mice for each genotype were used. Results are expressed as mean±SEM. *P<0.05; **P<0.01; ***P<0.001.
(TIF)

Figure S3 **Intestinal** ***Sirt1*** **deletion does not impact on intestinal stem cells.** **A,** Representative images of *In situ* hybridization using RNA probes for *Olfm4*. **B,** Crypt from *Sirt1^int−/−^*/*Lgr5-^EGFP-IRES-CreERT2^* and *Sirt1^L2/L2^/Lgr5^EGFP-IRES-CreERT2^* mice were isolated and GFP positive cells were detected by FACS analysis. No changes are observed in the percentage of GFP positive cells between the two groups (N = 6). **C,** Representative confocal images from *Sirt1^L2/L2^*/*Lgr5^EGFP-IRES-CreERT2^* and *Sirt1^int−/−^/Lgr5-^EGFP-IRES-CreERT2^* mice showing GFP^+^ ISC. Bar = 50 µm. **D,** Gene expression from the small intestine of *Sirt1^L2/L2^/Lgr5^EGFP-IRES-CreERT2^* and *Sirt1^int−/−^/Lgr5^EGFP-IRES-CreERT2^* mice shows no difference in mRNA levels of ISC genes (*Lgr5, Olfm4. Ascl2*). **E**, RTqPCR analysis of *Lgr5*, *Olfm4*, and *Prom1* mRNAs in proximal and distal small intestine of wild type and Sirt1^int−/−^ mice. For RTqPCR analysis *rps12* is used as reference. Results are expressed as mean±SEM. *P<0.05; **P<0.01; ***P<0.001.
(TIF)

Figure S4 **Proliferation assay and β-catenin detection in** ***Sirt1^int−/−^*** **and control mice.** **A,** BrDu^+^ cell staining and counts in *Sirt1^int−/−^* and control mice 2, 24, and 48 hours after injection. No differences are detected, highlighting the absence of a change in proliferation (N = 3 mice per time point; 5–10 fields per mouse/slide, 20–50 crypt/villi per field). Bar = 50 µm. **B,** Villi and crypts length in *Sirt1^int−/−^* and *Sirt1^L2/L2^* (N = 9 mice. 5–10 fields per mouse, 20–50 crypt/villi per field). **C,** Immunostaining of β-catenin in the ileum and colon of *Sirt1^int−/−^* and control mice. DAPI is used for nuclei staining. No differences are observed. **D,** Proteins from isolated crypts of *Sirt1^int−/−^* and *Sirt1^L2/L2^* were fractionated in order to detect β-catenin localization. No changes between the two genotypes are observed. Protein quantification was carried out trough ImageJ software (lower graph). Hsp90 is used as loading and fractionation control (Cyto = Cytoplasm; Nucl = nuclei).
(TIF)

File S1 **Supplemental Materials and Methods and four tables.**
(DOC)
