# Supplementary figures and images for: Loss of Sirt1 Function Improves Intestinal Anti-Bacterial Defense and Protects from Colitis-Induced Colorectal Cancer
Source: PLoS One. 2014 Jul 11;9(7):e102495. doi: 10.1371/journal.pone.0102495 (PMC4094521; doi:10.1371/journal.pone.0102495)

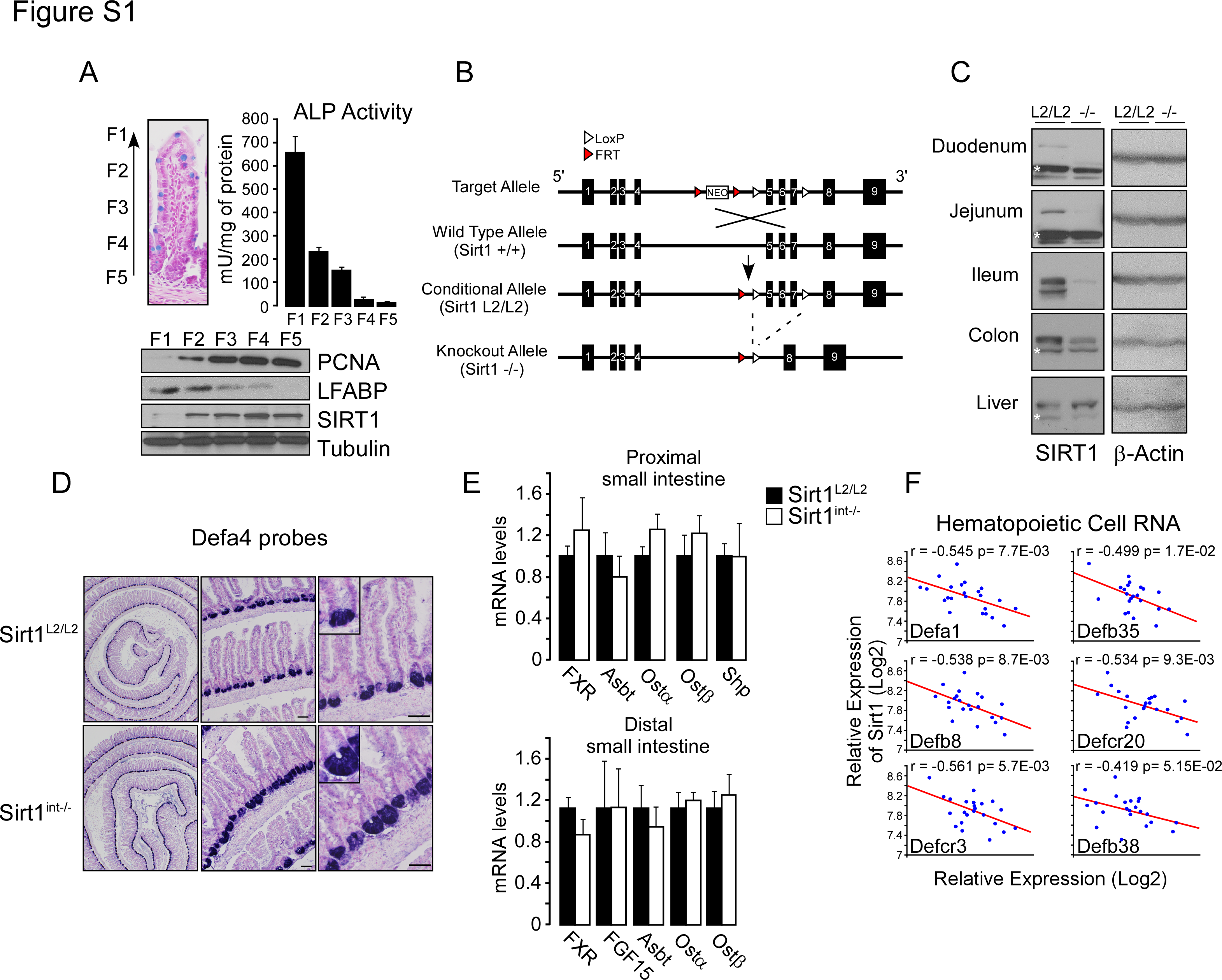

Supplement: Figure S1 — Generation and characterization of Sirt1int−/− mice. A, Villus/crypt fractionation shows a decrease in the alkaline phosphatase (ALP) activity from the top of the villus (F1) to the bottom (crypt, F5). SIRT1 shows a gradient increasing from the top to the bottom of the villus/crypt unit. PCNA and LFABP, respectively a proliferative and a differentiation marker, showed opposite protein distribution, confirming the validity of the fractionation protocol. Tubulin is the loading control. B, Schematic graph of the gene targeting strategy of exons 5–7 of the Sirt1 gene. C Western blot analysis of SIRT1 expression in intestine and liver of Sirt1int−/− and control mice showing the tissue-specific deletion of SIRT1. *Non-specific band. β-Actin is the loading control. D, In situ hybridization using Defa4 RNA probe to detect Paneth cells in Sirt1int−/− and Sirt1L2/L2 mice. E, RTqPCR analysis of bile acids transport and sensing mRNAs in proximal and distal small intestine of Sirt1L2/L2 and Sirt1int−/− mice (N = 6–8 mice). For RTqPCR analysis rps12 is used as reference. F, inverse correlation between expression of Sirt1 (y-axis) and mRNAs of each indicated defensin-related gene (x-axis) in hematopoietic cells of BXD mice strains (N = 22). Results are expressed as mean±SEM. (TIF) [file pone.0102495.s001.tif]

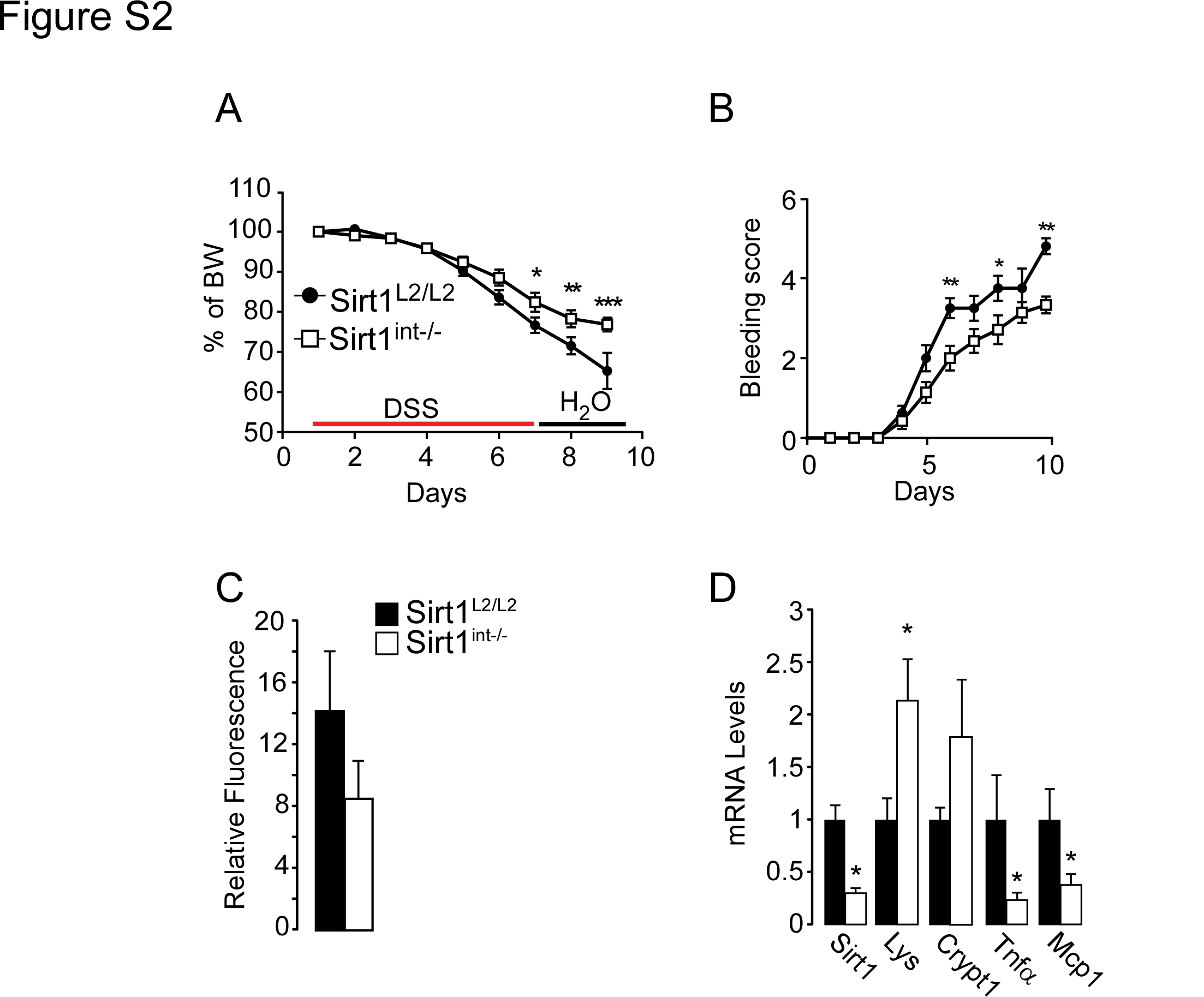

Supplement: Figure S2 — Intestinal Sirt1 deletion impacts on the development of colitis. A, Percentage of body weight loss observed during 7 days of 2% DSS treatment. B, Rectal bleeding score. Sirt1int−/− mice show significant less weight loss and a reduced bleeding score compared with wild type mice. Scoring details are in Supplemental Materials & Methods. ANOVA statistical analysis with Bonferroni post-hoc test was performed for each time point. C, In vivo intestinal permeability measurement. Sirt1int−/− mice show a reduced FITC-derived fluorescence in the blood suggesting less permeability, consequence of less inflammation. D, RT-qPCR analysis of lys, Crypt1, Tnfα, and Mcp1 mRNAs in the distal ileum of Sirt1int−/− and Sirt1L2/L2 after 2% DSS. Cyclophilin is used as reference. For the colitis experiment, 8 mice for each genotype were used. Results are expressed as mean±SEM. *P<0.05; **P<0.01; ***P<0.001. (TIF) [file pone.0102495.s002.tif]

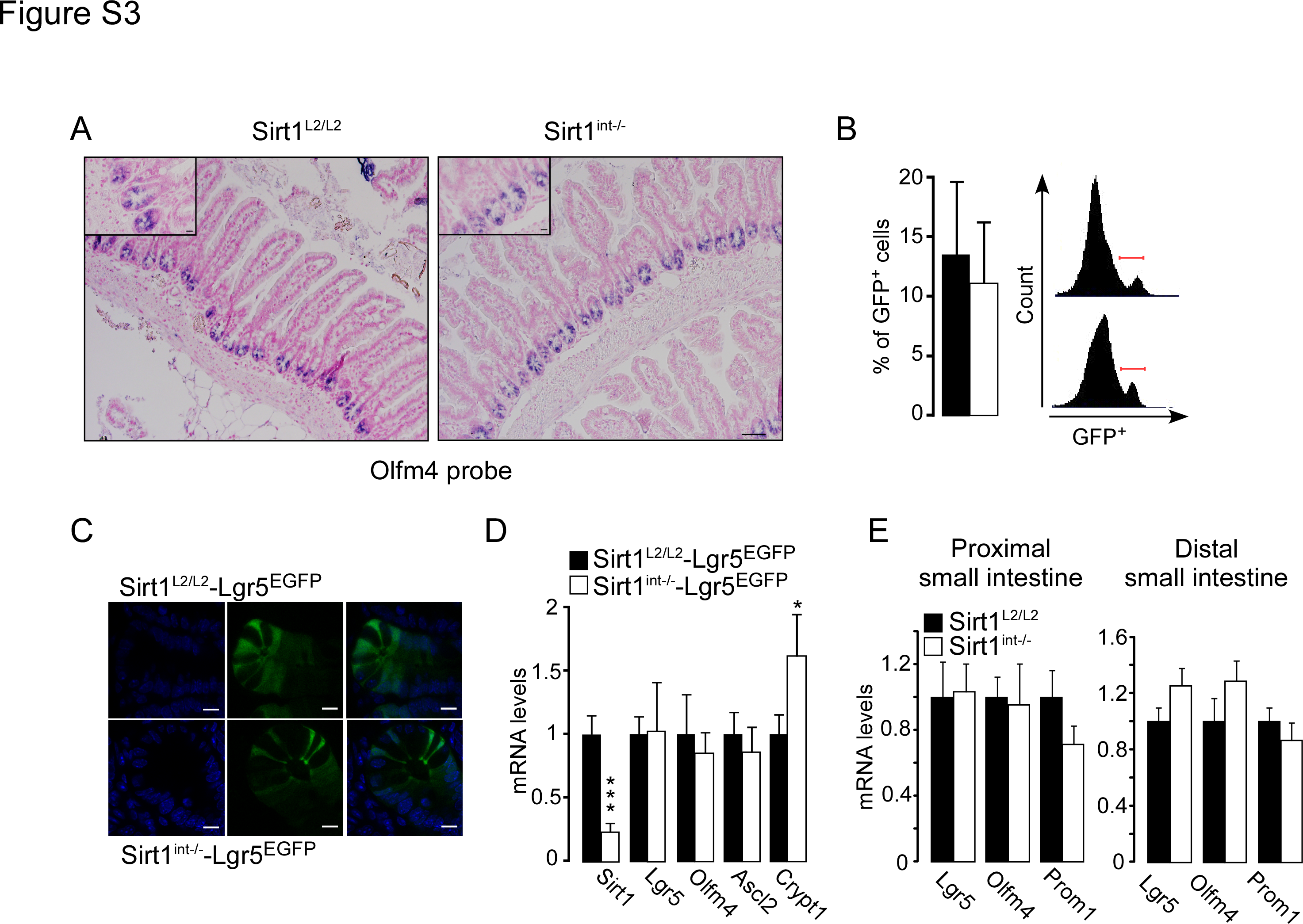

Supplement: Figure S3 — Intestinal Sirt1 deletion does not impact on intestinal stem cells. A, Representative images of In situ hybridization using RNA probes for Olfm4. B, Crypt from Sirt1int−/−/Lgr5-EGFP-IRES-CreERT2 and Sirt1L2/L2/Lgr5EGFP-IRES-CreERT2 mice were isolated and GFP positive cells were detected by FACS analysis. No changes are observed in the percentage of GFP positive cells between the two groups (N = 6). C, Representative confocal images from Sirt1L2/L2/Lgr5EGFP-IRES-CreERT2 and Sirt1int−/−/Lgr5-EGFP-IRES-CreERT2 mice showing GFP+ ISC. Bar = 50 µm. D, Gene expression from the small intestine of Sirt1L2/L2/Lgr5EGFP-IRES-CreERT2 and Sirt1int−/−/Lgr5EGFP-IRES-CreERT2 mice shows no difference in mRNA levels of ISC genes (Lgr5, Olfm4. Ascl2). E, RTqPCR analysis of Lgr5, Olfm4, and Prom1 mRNAs in proximal and distal small intestine of wild type and Sirt1int−/− mice. For RTqPCR analysis rps12 is used as reference. Results are expressed as mean±SEM. *P<0.05; **P<0.01; ***P<0.001. (TIF) [file pone.0102495.s003.tif]

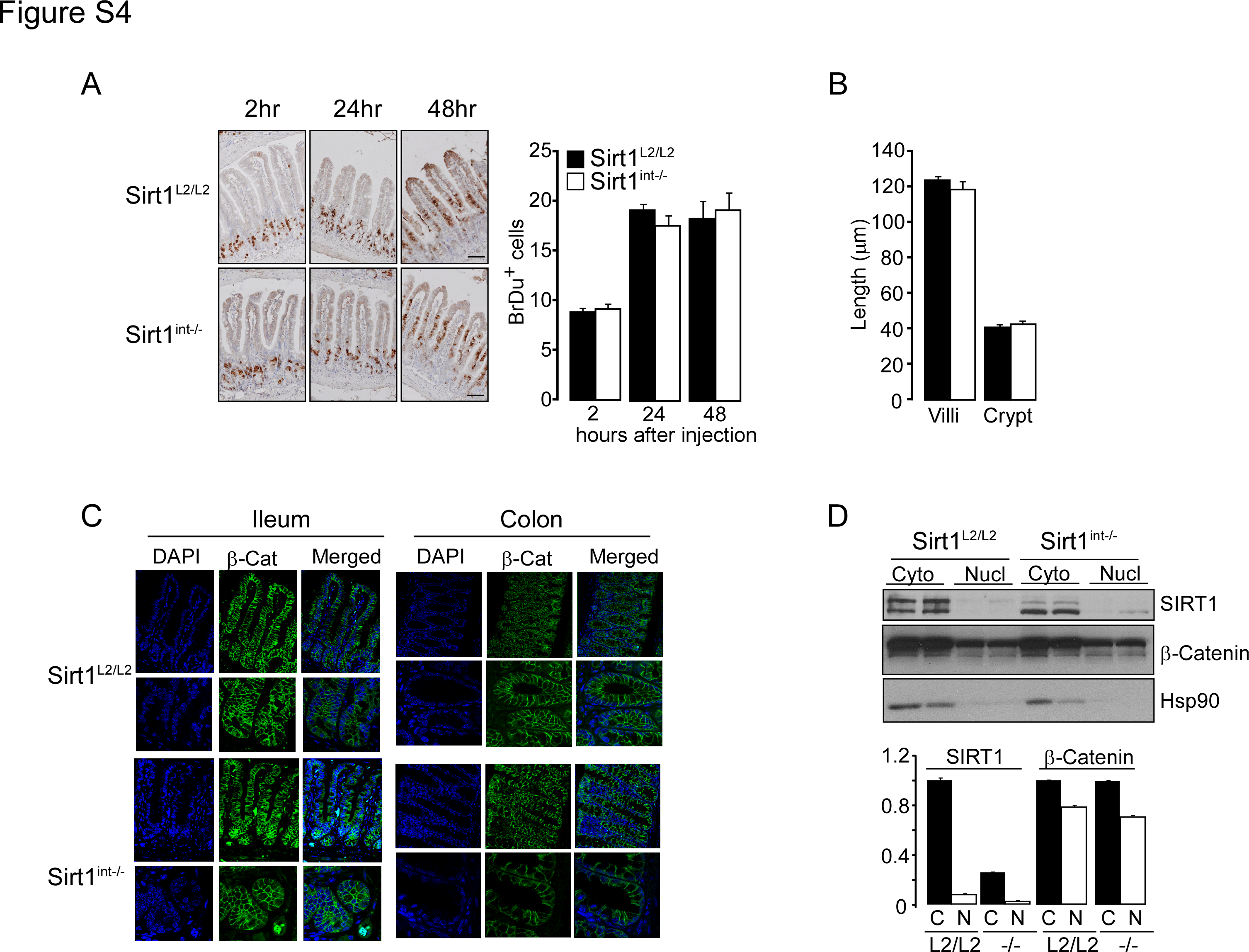

Supplement: Figure S4 — Proliferation assay and β-catenin detection in Sirt1int−/− and control mice. A, BrDu+ cell staining and counts in Sirt1int−/− and control mice 2, 24, and 48 hours after injection. No differences are detected, highlighting the absence of a change in proliferation (N = 3 mice per time point; 5–10 fields per mouse/slide, 20–50 crypt/villi per field). Bar = 50 µm. B, Villi and crypts length in Sirt1int−/− and Sirt1L2/L2 (N = 9 mice. 5–10 fields per mouse, 20–50 crypt/villi per field). C, Immunostaining of β-catenin in the ileum and colon of Sirt1int−/− and control mice. DAPI is used for nuclei staining. No differences are observed. D, Proteins from isolated crypts of Sirt1int−/− and Sirt1L2/L2 were fractionated in order to detect β-catenin localization. No changes between the two genotypes are observed. Protein quantification was carried out trough ImageJ software (lower graph). Hsp90 is used as loading and fractionation control (Cyto = Cytoplasm; Nucl = nuclei). (TIF) [file pone.0102495.s004.tif]
