# Supplementary material for: Loss of Sirt1 Function Improves Intestinal Anti-Bacterial Defense and Protects from Colitis-Induced Colorectal Cancer
Source: PLoS One. 2014 Jul 11;9(7):e102495. doi: 10.1371/journal.pone.0102495 (PMC4094521; doi:10.1371/journal.pone.0102495)
Supplement: File S1 — Supplemental Materials and Methods and four tables. (DOC) [file pone.0102495.s005.doc]

**Supporting Information**

**Loss of Sirt1 function improves intestinal anti-bacterial defense and protects from colitis-induced colorectal cancer.**

Giuseppe Lo Sasso^1^, Dongryeol Ryu^1^, Laurent Mouchiroud^1^, Samodha C. Fernando^2^, Christopher L. Anderson^2,3^, Elena Katsyuba^1^, Alessandra Piersigilli^1,4^, Michael O. Hottiger^5^, Kristina Schoonjans^1^, Johan Auwerx^1*^

**Short Title:** Loss of Sirt1 function improves intestinal physiology

*^1^Laboratory of Integrative and Systems Physiology, Ecole Polytechnique Fédérale de Lausanne, CH-1015 Lausanne, Switzerland. ^2^University of Nebraska, Department of Animal Science, Lincoln, NE 68583-0908, USA. ^3^School of Biological Sciences, University of Nebraska, Lincoln, NE, 68588, USA. ^4^Institute of Animal Pathology, University of Bern, Länggassstrasse 122, 3012 Bern, Switzerland. ^5^Institute of Veterinary Biochemistry and Molecular Biology, University of Zurich, Winterthurerstrasse 190, CH-8057 Zurich, Switzerland.*

*Correspondence: [admin.auwerx@epfl.ch](mailto:admin.auwerx@epfl.ch) (J.A.)

**Table S1, - List of primers used in qRT-PCR analyses.**

| **Mouse primers** | Forward | Reverse |
| --- | --- | --- |
| Sirt1 | GTCTCCTGTGGGATTCCTGA | ACACAGAGACGGCTGGAACT |
| Lysozyme | GTCACTGCTCAGGCCAAGGT | TAGCCAGCCATTCCATTCCT |
| Crypt1 | TCAAGAGGCTGCAAAGGAAGAGAAC | TGGTCTCCATGTTCAGCGACAGC |
| Crypt4 | CCAGGGGAAGATGACCAGGCTG | TGCAGCGACGATTTCTACAAAGGC |
| Defa-Rs | CACCACCCAAGCTCCAAATACACAG | ATCGTGAGGACCAAAAGCAAATGG |
| Klf4 | CAGACCAGATGCAGTCACAAGTC | TGGGCTCCTCTGGCAGG |
| Muc2 | ATGCCCACCTCCTCAAAGAC | GTAGTTTCCGTTGGAACAGTGAA |
| Lgr5 | TCCTAGAAGAGTTACGTCTTGCT | CCTTGGGAATGTGTGTCAAAGC |
| Olfm4 | GCCACTTTCCAATTTCAC | GAGCCTCTTCTCATACAC |
| Ascl2 | CCTCTCTCGGACCCTCTCTCAG | CAGTCAAGGTGTGCTTCCATGC |
| Prom1 | CACTCCTGACTGAAACACCAAAGC | TGCCATCCAGGTCTGAGAATGC |
| Spdef | GGACGGACGACTCTTCTGACAG | GCTCCTGATGCTGCCTTCTCC |
| Plau | GCGCCTTGGTGGTGAAAAAC | TTGTAGGACACGCATACACCT |
| Slug | TGGTCAAGAAACATTTCAACGCC | GGTGAGGATCTCTGGTTTTGGTA |
| Ccl6 | GCTGGCCTCATACAAGAAATGG | GCTTAGGCACCTCTGAACTCTC |
| Pax6 | AGTTCTTCGCAACCTGGCTA | GAGCCTCAATCTGCTCTTGG |
| Igfbp4 | AGAAGCCCCTGCGTACATTG | TTGTTGGGATGTTCGCTCTCA |
| Nrg3 | ACTCCCATACTTCCTGGTGA | GAGGAGCCAGTGAGGTAAGA |
| Tnfα | CATCTTCTCAAAATTCGAGTGACAA | TGGGAGTAGACAAGGTACAACCC |
| Cyclophilin | CAGGGGAGATGGCACAGGAG | CGGCTGTCTGTCTTGGTGCTCTCC |
| Rps12 | TAGGTTGGTGAGGGACGCT | CTAAGCGGTGCATCTGGTTC |
|  |  |  |
| ***C. elegans* primers** |  |  |
| Sir2.1 | TTCAAAACCACTCACCGCGA | CCAAGAATGTCGGTGGCTGA |
| Lys-1 | GGATCTGGAGCATTCGACACA | GCTGGGGAGGTAACCTGAAT |
| Lys-7 | GTCTCCAGAGCCAGACAATCCGG | CGGTCGTGATCTGATTCCAGTCG |
| Lys8 | ATTCGGCAGTCTTTGTCCGT | CCGGCTGTGGAGTCATGTAG |
| F53E10.4 | GCCTTATGTGTGCCAACTCG | TCGGCTGATAACGATCACGG |
| F55G11.2 | GGTGGCCACTTCCATCATCA | AGCTGGTAGAGACTTGCGAT |
| C32H11.12 | GGCATCCTTTGCTCTTGCAG | AGTCATCGTTCTGGAGCAATTC |
| F55G11.8 | GTCTGGTTTCCTCTTCCATCAGT | TTATCGTCTGCAGGGAAGGC |
| F01D5.5 | AAGCCTTCTGCCCAGTAACC | CACCCAGTTGACGCAGTTTG |
| F56D6.2 | TTTTGGAATGCAACCAGCGG | TTGTGGCGTATGGGCTGTAG |
| C17H12.8 | GTAGCGTTTTGGCTCCTTGC | TTGGCGGTGCGTTTACATTG |
| C29F3.7 | TGGAACATATTTCCCGGATGG | TCTGCGGCACGTTGATTTTG |
| K08D8.5 | GATTGGATACTGCGGCTGCT | AGACTCTCACGTTTGCATTGT |
| act-1 (b) | GCTGGACGTGATCTTACTGATTACC | GTAGCAGAGCTTCTCCTTGATGTC |
| Y45F10D.4 | GTCGCTTCAAATCAGTTCAGC | GTTCTTGTCAAGTGATCCGACA |

**Table S2, - Summary of Reads and alpha-diversity estimates. NS - no singletons**.

| **Sample** | **NS Reads** | **NS OTUs** | **Subsampled Chao1** | **Subsampled OTUs** | **Core Reads** | **Core OTUs** |
| --- | --- | --- | --- | --- | --- | --- |
| **Cecum Sirt1^L2/L2^-1** | 32996 | 537 | 477.10 | 219 | 32650 | 253 |
| **Cecum Sirt1^L2/L2^-2** | 40928 | 590 | 610.04 | 232 | 40574 | 297 |
| **Cecum Sirt1^L2/L2^-3** | 20923 | 322 | 496.53 | 181 | 20785 | 199 |
| **Cecum Sirt1^L2/L2^-4** | 57781 | 555 | 306.78 | 165 | 56585 | 223 |
| **Cecum Sirt1^int-/-^-5** | 16018 | 386 | 678.33 | 249 | 15831 | 215 |
| **Cecum Sirt1^int-/-^-6** | 25626 | 530 | 612.48 | 252 | 25312 | 267 |
| **Cecum Sirt1^int-/-^-7** | 18834 | 440 | 653.53 | 261 | 18589 | 234 |
| **Cecum Sirt1^int-/-^-8** | 27087 | 502 | 677.43 | 236 | 26746 | 229 |
| **Cecum Sirt1^int-/-^-9** | 25958 | 488 | 558.62 | 243 | 25476 | 195 |
| **Colon Sirt1^L2/L2^-1** | 46182 | 616 | 677.05 | 222 | 45762 | 278 |
| **Colon Sirt1^L2/L2^-2** | 48317 | 1045 | 886.98 | 369 | 46825 | 336 |
| **Colon Sirt1^L2/L2^-3** | 14699 | 329 | 627.13 | 228 | 14562 | 205 |
| **Colon Sirt1^L2/L2^-4** | 29924 | 301 | 307.53 | 137 | 29658 | 156 |
| **Colon Sirt1^int-/-^-5** | 11559 | 307 | 528.64 | 238 | 11436 | 196 |
| **Colon Sirt1^int-/-^-6** | 16047 | 501 | 735.73 | 307 | 15783 | 270 |
| **Colon Sirt1^int-/-^-7** | 52382 | 993 | 1081.81 | 301 | 51469 | 358 |
| **Colon Sirt1^int-/-^-8** | 65420 | 1062 | 870.00 | 330 | 63802 | 301 |
| **Colon Sirt1^int-/-^-9** | 26922 | 453 | 545.62 | 208 | 26546 | 211 |
| **AOM Sirt1^L2/L2^-10** | 7857 | 176 | 415.08 | 176 | 7778 | 106 |
| **AOM Sirt1^L2/L2^-11** | 11622 | 279 | 409.08 | 226 | 11412 | 173 |
| **AOM Sirt1^L2/L2^-12** | 32254 | 539 | 683.75 | 263 | 31525 | 290 |
| **AOM Sirt1^int-/-^-13** | 37939 | 602 | 467.49 | 239 | 37355 | 271 |
| **AOM Sirt1^int-/-^-14** | 11983 | 413 | 675.14 | 309 | 11769 | 238 |
| **AOM Sirt1^int-/-^-15** | 59851 | 387 | 317.07 | 122 | 59584 | 214 |
|  |  |  |  |  |  |  |
| **Total** |  | 3527 | - | 2207 | 727814 | 607 |

**Table S3, -** Top 20 most statistically significant OTUs with total abundance greater than 20 reads belonging to the core measurable microbiome (CMM)

| **OTU ID** | **P-Value** | **RDP Classifcation** | **Closest NCBI Hit** | **E-value** | **Percent Identity** | **Accession #** |
| --- | --- | --- | --- | --- | --- | --- |
| **65** | 2.60E-07 | Family - Rikenellaceae | *Alistipes putredinis* | 1E-64 | 95% | NR_025909.1 |
| **145** | 1.31E-05 | Family - S24-7 | *Barnesiella intestinihominis* | 2E-33 | 86% | NR_041668.1 |
| **20** | 6.05E-04 | Genus - Bacteroides | *Bacteroides eggerthii* | 8E-60 | 93% | NR_040864.1 |
| **23** | 1.02E-03 | Family - S24-7 | *Barnesiella intestinihominis* | 6E-36 | 83% | NR_041668.1 |
| **9** | 1.87E-03 | Family - S24-7 | *Barnesiella intestinihominis* | 4E-51 | 89% | NR_041668.1 |
| **112** | 2.07E-03 | Family - Lachnospiraceae | *Clostridium populeti* | 4E-57 | 97% | NR_026103.1 |
| **19** | 2.42E-03 | Genus - Oscillospira | *Flavonifractor plautii* | 5E-56 | 94% | NR_043142.1 |
| **26** | 2.54E-03 | Family - S24-7 | *Barnesiella intestinihominis* | 1E-38 | 83% | NR_041668.1 |
| **64** | 3.92E-03 | Family - S24-7 | *Barnesiella intestinihominis* | 2E-43 | 86% | NR_041668.1 |
| **81** | 4.00E-03 | Genus - Bacteroides | *Bacteroides fragilis* | 4E-57 | 91% | NR_074839.1 |
| **66** | 4.27E-03 | Family - Lachnospiraceae | *Clostridium populeti* | 2E-60 | 98% | NR_026103.1 |
| **43** | 4.38E-03 | Genus - Bacteroides | *Bacteroides eggerthii* | 5E-49 | 91% | NR_040864.1 |
| **214** | 5.06E-03 | Genus - Prevotella | *Prevotella dentalis* | 3E-52 | 93% | NR_102481.1 |
| **12** | 5.56E-03 | Genus - Prevotella | *Prevotella dentalis* | 4E-64 | 94% | NR_102481.1 |
| **39** | 6.63E-03 | Genus - Bacteroides | *Bacteroides eggerthii* | 5E-49 | 91% | NR_102481.1 |
| **77** | 7.41E-03 | Genus - Bacteroides | *Bacteroides eggerthii* | 1E-64 | 95% | NR_102481.1 |
| **59** | 8.20E-03 | Family - Lachnospiraceae | *Clostridium populeti* | 4E-42 | 97% | NR_026103.1 |
| **38** | 1.02E-02 | Order - Clostridiales | *Clostridium sporosphaeroides* | 1E-35 | 95% | NR_044835.1 |
| **5** | 1.31E-02 | Family - S24-7 | *Persicitalea jodogahamensis* | 5E-31 | 79% | NR_041525.1 |
| **1** | 1.49E-02 | Family - Lachnospiraceae | *Clostridium populeti* | 3E-58 | 97% | NR_026103.1 |

**Table S4. Most statistically significant OTUs with total abundance greater than 20 reads belonging to the core measurable microbiome (CMM) before and after AOM treatment in both Sirt1^L2/L2^ and Sirt1^int-/-^.**

| **OTU ID** | **P-Value** | **RDP Classifcation** | **Closest NCBI Hit** | **E-value** | **Percent Identity** | **Accession #** |
| --- | --- | --- | --- | --- | --- | --- |
| **Sirt1^L2/L2^** |  |  |  |  |  |  |
| **4** | 2.47E-03 | Family - Lachnospiraceae | *Clostridium saccharolyticum* | 2.00E-47 | 95% | NR_102852.1 |
| **128** | 7.50E-03 | Family - Helicobacteraceae | *Helicobacter pullorum* | 7.00E-53 | 98% | NR_043053.1 |
| **38** | 8.50E-03 | Order - Clostridiales | *Clostridium sporosphaeroides* | 1.00E-35 | 95% | NR_044835.1 |
| **44** | 1.29E-02 | Family - S24-7 | *Barnesiella intestinihominis* | 3.00E-47 | 87% | NR_041668.1 |
| **13** | 2.09E-02 | Family - Lachnospiraceae | *Clostridium populeti* | 6.00E-55 | 94% | NR_026103.1 |
| **11** | 3.16E-02 | Genus - Desulfovibrio | *Desulfovibrio desulfuricans* | 4.00E-64 | 94% | NR_074858.1 |
| **Sirt1^int-/-^** |  |  |  |  |  |  |
| **12** | 4.03E-03 | Genus - Prevotella | *Prevotella dentalis* | 4.00E-64 | 94% | NR_102481.1 |
| **9** | 8.89E-03 | Family - S24-7 | *Barnesiella intestinihominis* | 4.00E-51 | 89% | NR_041668.1 |
| **92** | 1.74E-02 | Phylum - Bacteroidetes | *Flexibacter ruber* | 2.00E-16 | 78% | NR_040916.1 |
| **149** | 2.55E-02 | Family - S24-7 | *Barnesiella intestinihominis* | 1.00E-31 | 80% | NR_041668.1 |

**Materials and Methods**

**16S rRNA amplicon library preparation** – 16S rRNA gene amplicon libraries of the V3 region were prepared using custom barcoded universal fusion primers as described using a barcoded 518R primer. Multiplex barcodes were used during sequencing to preserve sample identity and was demultiplexed using the barcode sequence after sequencing to obtain reads corresponding to each sample. Briefly, genomic DNA from tissue and feces was extracted using Nucleospin Tissue (Macherey-Nagel, Düren, Germany) or QIAmp DNA Stool (Qiagen, Hilden, Germany) respectively. A 25μl PCR reaction contained 0.5 Units of Terra DNA polymerase (Clontech Laboratories, Mountain view, CA), 1X reaction buffer, 200 μM dNTPs (New England Biolabs, Ipswich, MA), 200 nM of each primer, 0.1 µg/µL BSA (New England Biolabs, Ipswich, MA), and 50-100 ng of nucleic acid template or no-template control. The cycling conditions included an initial denaturation of 98°C for 3 min, followed by 30 cycles of 98°C for 30 s, 52°C for 30 s, and 68°C for 1 min; and a final extension of 68°C for 2 min. Amplified PCR fragments were pooled based on copies of the 16S rRNA gene calculated using qPCR of the V3 amplicons generated. Briefly, the qPCR reaction was performed using a nested 518R primer 5’-ATTACCGCGGCTGCTGGCAC-3’ and the universal 341F primer. The 15μl reaction contained, 1X SYBR green master mix, 200 nM of each primer, and 2ng of PCR product. The cycling conditions included an initial denaturation of 95°C for 10 min, followed by 30 cycles of 95°C for 30 s, 52°C for 30 s, and 72°C for 1 min; followed by a melting curve. The pooled PCR products were purified on a 2.0% E-Gel SizeSelect Gel (Qiagen, Valencia, CA), and PCR product size, quality, and quantity were evaluated using a Bioanalyzer 2100 (Agilent, Palo Alto, CA) using a DNA1000 LabChip and used for Ion Torrent sequencing.

**Sequencing and Quality control** – Sequencing was carried out on the Ion Torrent Personal Genome Machine with a 200 Sequencing Kit v2 on a 316 chip according to manufacturer’s protocols. The emulsion PCR reaction (11pM of pooled DNA) was performed using the Ion PGM Template OT2 400 reaction kit using the OneTouch 2 System according to manufacturer’s protocols. Methods used for emPCR, bead deposition, and sequencing on the PGM was as described by the manufacturer. Initial quality control of sequences generated was performed using the Torrent Suite Software version 3.6.2, which included trimming of the 3’ end of sequences that dropped below the average Q15 score over a 30 bp window and removing sequences with unidentified bases (N). Resulting sequences were downloaded from the Torrent Suite and demultiplexed within the QIIME software package. Sequences were allowed a default mismatch of 1.5 errors to the barcode. Following demultiplexing, universal primers used for sequencing were removed, allowing 1 mismatch in the 5’ (518R) primer and 2 in the 3’ reverse primer (341F). Sequences were trimmed to a minimum and maximum length of 80 and 177 bp, respectively. Quality trimmed sequences were reverse complemented for bioinformatics community analysis.

**OTU picking** – The mothur 454 SOP was used as a framework to analyze the resulting sequences with a few key exceptions. Sequences were not processed using flowgrams and unique sequences were aligned in RDP. Subsequently, sequences not matching the aligned V3 region were removed, and pre-clustered. De novo and reference based methods were utilized to remove chimeras with the mothur (v.1.31.2) implemented version of UCHIME before calculation of a distance matrix (dist.seqs) and subsequent clustering based on the average neighbor algorithm. Files resulting from the make.shared, bin.seqs, and get.oturep commands were reformatted with custom Perl scripts to be suitable for OTU table generation and taxonomy assignment in MacQIIME (QIIME version 1.7) for downstream analysis.

**Microbial Community Analysis** – Taxonomy was assigned to each OTU using representative sequences using the RDP “CLASSIFIER” algorithm trained on the GreenGenes taxonomy release (database 12_10) using default parameters within the QIIME package. OTUs with a single read were subsequently removed as they were likely sequencing errors missed during quality control or represent rare lineages not of biological significance for the current study. A Core Measurable Microbiome (CMM) was defined for each of the defined treatment groups. An OTU was considered as part of the CMM if a specific OTU was present in 75% of the *Sirt1^L2/L2^* mice (3 out of 4 samples), 60% of the *Sirt1^int-/-^* mice (3 out of 5 samples), 66% of the *Sirt1^L2/L2^* mice treated with AOM (2 out of 3 samples), and 66% of the *Sirt1^int-/-^* mice treated with AOM (2 out of 3 samples). A CMM is defined to focus further analyses on OTUs that are most likely to represent the treatment group. Further, this serves to likely remove many remaining spurious OTUs.

**Statistical Analysis for microbial community analysis** – All samples were sub-sampled to the same number of reads before statistical analysis to prevent unequal sampling biasing analysis. An unweighted UniFrac matrix was used for community clustering, and principal coordinate analysis (PCA) with built in QIIME commands. Phylogenetic information for UniFrac was inferred from a phylogentic tree constructed in clearcut from a phylip distance matrix created using mothur dist.seqs command. In addition, differences in microbial community structure among phenotypes were explored using Permutational multivariate analysis of variance using PERMANOVA, and analysis of similarity using ANOSIM within the Fathom package in matlab. PERMANOVA and ANOSIM were performed using the unweighted unifrac distance matrix. The significance of OTU association with the SIRT1 genotype was tested by analysis of variance (ANOVA) implemented within QIIME using the model described above. The indicator OTU abundance was log transformed, standardized and plotted within matlab using the “HeatMap” command. P-values < 0.05 was used for significance.

**Supplemental References**

1. Whiteley, AS, Jenkins S, Waite I, Kresoje N, et al. (2012). Microbial 16S rRNA Ion Tag and community metagenome sequencing using the Ion Torrent (PGM) Platform. J Microbiol Methods; **91:** 80-88.

2. Caporaso, JG, Kuczynski J, Stombaugh J, Bittinger K, et al. (2010). QIIME allows analysis of high-throughput community sequencing data. Nat Methods; **7:** 335-336.

3. Cole, JR, Wang Q, Cardenas E, Fish J, et al. (2009). The Ribosomal Database Project: improved alignments and new tools for rRNA analysis. Nucleic Acids Res; **37:** D141-145.

4. Nawrocki, EP, Kolbe DL & Eddy SR (2009). Infernal 1.0: inference of RNA alignments. Bioinformatics; **25:** 1335-1337.

5. Schloss, PD, Westcott SL, Ryabin T, Hall JR, et al. (2009). Introducing mothur: open-source, platform-independent, community-supported software for describing and comparing microbial communities. Appl Environ Microbiol; **75:** 7537-7541.

6. Edgar, RC, Haas BJ, Clemente JC, Quince C, et al. (2011). UCHIME improves sensitivity and speed of chimera detection. Bioinformatics; **27:** 2194-2200.

7. Wang, Q, Garrity GM, Tiedje JM & Cole JR (2007). Naive Bayesian classifier for rapid assignment of rRNA sequences into the new bacterial taxonomy. Appl Environ Microbiol; **73:** 5261-5267.

8. Lozupone, C, Lladser ME, Knights D, Stombaugh J, et al. (2011). UniFrac: an effective distance metric for microbial community comparison. ISME J; **5:** 169-172.

9. Evans, J, Sheneman L & Foster J (2006). Relaxed neighbor joining: a fast distance-based phylogenetic tree construction method. J Mol Evol; **62:** 785-792.
